# Following Multiple Failed Reconstructions of a Distal Femur Fracture, Osseous Union Achieved After Superficial Femoral Artery Endarterectomy

**DOI:** 10.31486/toj.24.0058

**Published:** 2025

**Authors:** Shane Johns, William Curtis, Rick Gehlert

**Affiliations:** ^1^ University of New Mexico School of Medicine, Albuquerque, NM; ^2^ Department of Orthopaedics & Rehabilitation, University of New Mexico School of Medicine, Albuquerque, NM

**Keywords:** *Femoral fractures–distal*, *fracture fixation–internal*, *open fracture reduction*, *orthopedic fixation devices*, *orthopedic procedures*

## Abstract

**Background:**

Nonunion of a distal femur fracture is a serious complication in which bone healing ceases or fails to resolve, often necessitating further surgical intervention. Poor blood supply and unstable fixation have been identified as contributing factors to osseous nonunion. In this case, we highlight a challenging femur fracture that achieved osseous union only after resolution of a superficial femoral artery occlusion via endarterectomy that improved blood flow to the fracture site.

**Case Report:**

A 54-year-old male was involved in a high-speed motor vehicle accident that resulted in a left distal femur fracture. The patient underwent multiple reconstructive procedures that were complicated by hardware failure and recurrent nonunion. Prior to the sixth reconstruction, a superficial femoral artery occlusion was discovered and addressed with endarterectomy. The sixth and final procedure resulted in osseous union and stable fixation of the femur fracture.

**Conclusion:**

A missed superficial femoral artery occlusion likely contributed to the delay in achieving osseous union of a traumatic comminuted distal femur fracture.

## INTRODUCTION

Nonunion, a severe complication of distal femur fractures, occurs when bone healing halts or fails to resolve. While long bone nonunion is a known complication of fracture fixation, the specific definition of nonunion is debated, and a general consensus on the definition has yet to be determined.^1^ However, nonunion is often defined as the inability of a fracture to achieve osseous union in the expected amount of time, generally necessitating surgical intervention.^2,3^ Unstable fixation and inadequate blood supply have both been identified as common contributors to nonunion.^4-6^ Additionally, primary fixation of a distal femur fracture with a single locking plate has an up to 19% chance of resulting in nonunion.^7^

We report a case of multiple failed attempts to reconstruct a distal femur fracture that ultimately formed osseous union after a superficial femoral artery occlusion was addressed with an endarterectomy.

## CASE REPORT

**Figure 1.**
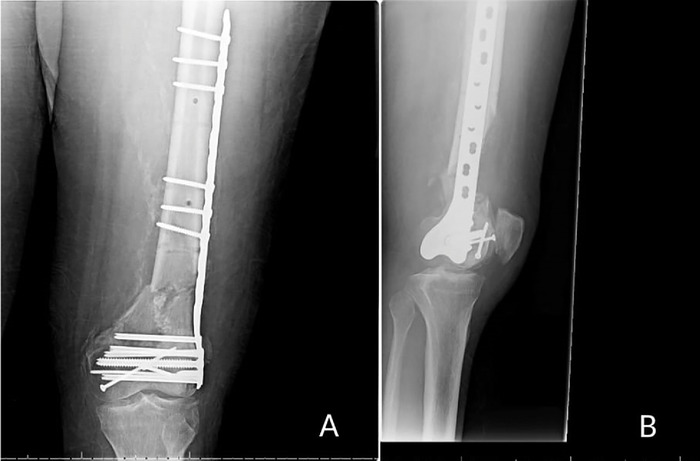
(A) Anteroposterior and (B) lateral radiographic films show the left femur following the first reconstruction after the motor vehicle collision in 2012.

A 54-year-old male with a medical history significant for a 22 pack-year smoking history, hypertension, and a body mass index (BMI) of 30 kg/m^2^ was involved in a high-speed motor vehicle collision in 2012. The collision resulted in multiple traumatic orthopedic injuries, including a comminuted distal left femur fracture that was treated at an outside hospital with open reduction and internal fixation (ORIF) of the articular elements using a lateral locking plate (Figure 1). Upon discharge from the outside facility, the patient was recommended to follow up with our institution (senior author RG) where the patient received the remainder of his orthopedic care.

**Figure 2.**
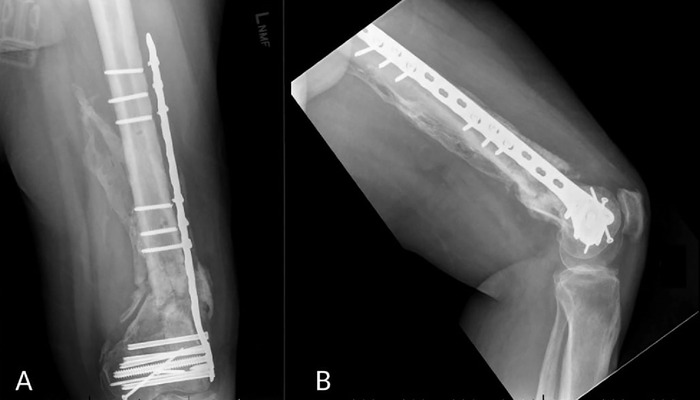
(A) Anteroposterior and (B) lateral radiographic films show hardware failure of the proximal plate with supracondylar nonunion occurring 16 months after the first open reduction and internal fixation procedure. This hardware failure was the first of five.

Despite appropriate initial fixation and follow-up imaging consistent with interval healing of the fracture, the patient later developed a nonunion at the supracondylar aspect of the fracture with proximal screw breakage, varus drift, and shortening diagnosed on subsequent radiographic imaging (Figure 2). The patient returned to the operating room (OR) approximately 16 months after the original ORIF to address the missing bone at the site of the femur fracture. The second ORIF included decortication of the nonunion site with a burr and revision fixation with a 14-hole locking distal femur plate (DePuy Synthes) in almost exactly the same location as the removed plate. Heterotopic bone that had grown over the failed plate was harvested and utilized for morselized bone autograft. Good fixation was achieved.

Despite achieving satisfactory fixation of the fracture and repeat radiographs demonstrating interval healing at postoperative appointments, the patient again experienced a nonunion with hardware failure and returned to the OR for a third procedure, approximately 16 months after the second ORIF. The third procedure required removal of the previous hardware, reconstruction of the distal femur nonunion site with a titanium mesh cage, use of allograft stem cell matrix and recombinant human bone morphogenetic protein (BMP), and revision fixation of the distal femoral shaft utilizing a 14-hole locking plate (DePuy Synthes). Following this procedure, the patient was evaluated for nutritional deficiency with laboratory testing of albumin, total protein, vitamin D, and iron. The laboratory results did not demonstrate any gross dietary inadequacies.

Two weeks following the third ORIF, the patient was seen in the emergency department for increased drainage from one of his surgical incisions, which resolved after a course of antibiotics. Patient history, examinations, and imaging at postoperative appointments following the third reconstruction were consistent with interval healing of the fracture site, and the patient was able to bear weight on the left leg with the assistance of crutches.

The patient returned to his day job as an automobile mechanic. However, he began to experience worsening pain approximately 15 months postoperatively. The patient was reevaluated in the clinic, and repeat imaging of the left femur demonstrated a broken distal femoral plate as well as increased lucency at the distal femur fracture site. Significant callus and a compressed allograft bone cage indicated some interval healing.

The patient's fourth reconstruction was performed 19 months after the third procedure. This procedure consisted of removing old hardware, recanalizing the distal femur, harvesting 25 to 30 cm^3^ of bone autograft, placing a tantalum cone to prevent further shortening, performing revision fixation with a custom distal femur locking plate (Smith & Nephew plc), and placing prepared recombinant human BMP strips over the bone graft to further stimulate healing.

After the fourth procedure, the patient did well, and imaging at postoperative appointments demonstrated continued interval healing of the femur. However, approximately 11 months postoperatively, the patient was working on a vehicle when he twisted on the left lower extremity and felt an immediate cracking sensation followed by severe pain near the site of the healing left femur injury. He was reevaluated in the clinic, and imaging demonstrated failure of the hardware. The decision was made to again return to the OR.

During the patient's fifth surgery, obvious callus formation was encountered, indicating some level of healing. The fifth procedure included removal of the old hardware and revision fixation using a non-contact bridging distal femur plate (Zimmer Biomet) similar in length to the removed hardware. The plate was then pressed into approximately 5 to 7 degrees of valgus and the location was selected in an effort to preserve the healing that had already taken place.

Approximately 12 weeks after the fifth surgery, the patient began to complain of increasing pain and dysfunction. He obtained imaging from an outside facility that demonstrated failure of the left femur hardware once again. At the time, the patient was receiving treatment at an outside facility for a chronic left foot wound involving the second digit. The recommendation was for the patient to defer further intervention on his left femur until the foot wound completely healed. The patient was also seen by a cardiologist at the outside facility who ordered an ankle brachial index (ABI) test to assess the perfusion in the patient's left extremity. The left ABI of 0.56 (reference range, 1.0-1.4) demonstrated moderate to severe peripheral artery disease, confirming the suspicion that the patient had poor vascular perfusion of the left lower extremity. The left toe brachial index (TBI) was 0.29 (reference value, ≥0.7), demonstrating poor perfusion of the left foot. Additionally, the patient was diagnosed with a cardiac arrythmia and placed on chronic anticoagulation therapy. An atherosclerotic occlusion involving the distal left superficial femoral artery in the region of the adductor canal was identified on angiography, and an endarterectomy was recommended prior to any further orthopedic intervention. In October 2018, a left superficial femoral artery endarterectomy was performed to restore perfusion to the lower limb. Following the procedure, left ABI and TBI improved to 0.77 and 0.36, respectively.

Following the resolution of the chronic foot wound, the patient returned to our orthopedic clinic seeking further treatment options for the femur. The sixth and final reconstruction of the patient's left femur was performed just over 2 years after the fifth reconstruction and more than 7 years after the initial injury. The previous hardware was removed, except for the tantalum cone that had achieved good osseous ingrowth. A new plate was fixed to the fracture site, and autograft from the patient's superior patella was used to address any bone loss. Anecdotally, increased bleeding was noted during this procedure in comparison to prior surgeries, leading to the decision to administer tranexamic acid that the patient did not receive in previous procedures.

**Figure 3.**
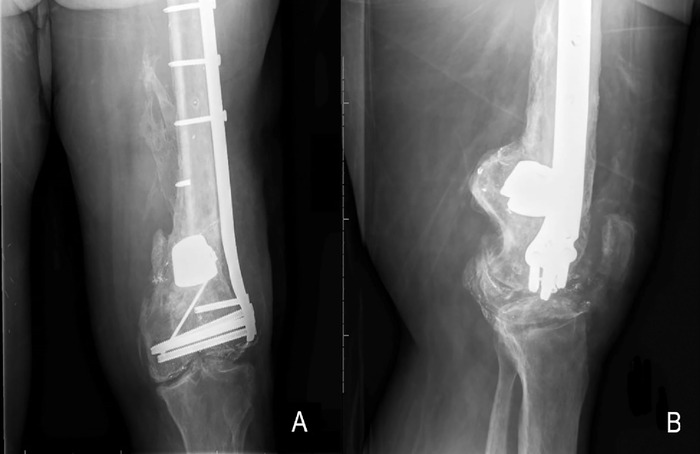
(A) Anteroposterior and (B) lateral radiographic films at final evaluation, more than 7 years after the patient's initial injury and after superficial femoral artery endarterectomy, show osseous healing of the distal femur with no signs of hardware loosening.

**Figure 4.**
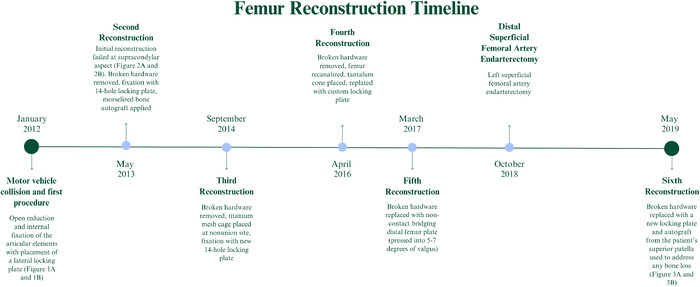
Timeline demonstrates the series of operations from the initial injury in 2012 to the final reconstruction in 2019.

The patient's final evaluation occurred 19 months following the sixth reconstruction. At that time, imaging demonstrated stable fixation with complete union of the femur fracture (Figure 3). Since his last operation, the patient has been able to ambulate with forearm crutches, although his motion and function are limited by posttraumatic tricompartmental arthritis of the left knee and right rotator cuff tear. On final examination, the patient had complete extension of the knee and 85 degrees of passive flexion without pain. Considering the patient's complex medical history and extensive surgical history of the left lower extremity, he was content with this outcome and has been able to return to work. The total time from the initial injury to the final procedure was more than 7 years. Figure 4 presents a timeline of the patient's femur reconstruction procedures.

## DISCUSSION

This case presents a 54-year-old male for whom multiple attempts at distal femur fracture fixation failed until a superficial femoral artery occlusion was discovered and addressed with endarterectomy at an outside hospital. Osseous union was successful only after the endarterectomy. While distal femur ORIF procedures are fairly routine for patients with orthopedic trauma, the literature reports continued challenges with nonunion. As stated earlier, distal femur fractures treated with single plate fixation ORIF have up to a 19% chance of resulting in nonunion.^7^ Perhaps the most challenging part of this patient's treatment is that the fixated femur often failed more than 12 months into postoperative recovery, effectively restarting the patient's recovery each time.

Determining and mitigating any factors contributing to nonunion can improve patient outcomes and reduce the likelihood of complications requiring multiple procedures. Factors associated with nonunion include surgical technique; elevated BMI; open fractures; infection; bone loss; and, perhaps most important, vascularity that can be impacted by multiple variables including tobacco use, diabetes, trauma, atherosclerosis, embolism, thrombosis, and other causes of obstruction.^4^ Traumatic orthopedic surgery does not always allow for optimal mitigation of nonunion risk factors such as BMI and longstanding nicotine use, and our patient's medical/social history was significant for a BMI of 30 kg/m^2^, a 22 pack-year smoking history, and hypertension. Local measures taken throughout this case to improve blood flow included debridement of scar tissue back to viable muscle and freshening bone ends with a high-speed burr. Whether these attempts to improve blood flow were efficacious is unclear, considering that these procedures were done in the setting of an unknown proximal vascular injury that was only later identified.

During the evaluation for a cause of the patient's nonunion, cultures were obtained to assess for low-grade infection. Cultures from the surgical site and relevant blood work did not demonstrate any evidence of infection throughout this case. The patient had an episode of increased wound drainage following the third operation that was treated with a course of outpatient antibiotics, but the authors of this case do not have any suspicion that infection played a role in the difficulty of obtaining union.

Throughout his treatment, the patient attended numerous physical therapy sessions with strict instructions following each femur reconstruction. Postoperative instructions and recommendations included toe touch-down weight-bearing status, followed by progressing to bearing more weight on the extremity as tolerated. Because of financial stress and the prolonged duration of his treatment, the patient was compelled to return to his physically demanding job as an automobile mechanic, which likely complicated his recovery.

The vascular aspects of this case highlight the importance of the superficial femoral artery, its branches, and the perfusion of the distal femur. The superficial femoral artery courses down the medial posterior thigh until it passes through the Hunter canal of the adductor magnus muscle to become the popliteal artery.^8^ This region of the adductor canal is a common location for the superficial femoral artery to develop atherosclerosis, hypothesized to occur because of compression of the vessel at this site.^9^ The distal femur primarily receives blood flow from the descending genicular artery and the left and right genicular arteries, which are branches of the superficial femoral artery and popliteal artery, respectively.^10^ Additional blood supply to the distal femur includes the descending branch of the lateral circumflex artery, a branch of the deep femoral artery. Finally, the popliteal artery continues down the posterior knee until it branches into the anterior tibial, posterior tibial, and fibular arteries. These arteries carry blood to the lower segment of the leg, and perfusion is often evaluated at these sites by palpating the dorsalis pedis and tibialis posterior pulses.

The surgical treatments used throughout this case were carefully reviewed to evaluate for a potential cause of the patient's repetitive failed union. Surgical treatment of a distal femur fracture depends on the degree of displacement and fracture pattern. The 2 primary treatment options for an initial ORIF of a distal-third femur fracture are intramedullary nail and lateral plate fixation. For simple fracture patterns, the literature does not currently demonstrate that one treatment option leads to significant improvement in achieving union, joint motion, patient satisfaction, or decreased mortality than the other.^11^ However, for comminuted fractures, lateral plate fixation is often recommended to buttress the fracture, bridge the comminution, and achieve adequate fixation.^12-16^ Additionally, lateral plate fixation is currently the recommended technique for revision of femoral fracture nonunion.^16^ The initial surgical technique and subsequent revisions used in this case were appropriate and were likely not contributing factors to the multiple nonunions the patient experienced.

Although we may never conclusively ascertain whether the superficial femoral artery reperfusion procedure directly contributed to the resolution of our patient's bony nonunion, ample evidence supports the necessity of sufficient blood flow for union to occur. Bone healing and consolidation depend on the delivery of oxygen, growth factors, and removal of metabolic waste through an adequate circulatory system.^17^ Our patient's 22 pack-year tobacco smoking history likely also impaired blood flow to the fracture site at baseline, as nicotine is a well-known risk factor for nonunion.^18-20^ Perfusion of the fracture site was undoubtedly compromised by the atheroma occluding the superficial femoral artery. We hypothesize that the patient had chronic vascular compromise at baseline, and that the endarterectomy improved blood flow sufficiently to allow fracture healing.

We are not aware of any other research reporting a similar clinical presentation of multiple nonunions that resolved following a reperfusion procedure. However, developing research in blood supply and its relationship to achieving bone union may help elucidate treatment options for chronic nonunions. Menger et al evaluated new imaging techniques to assess nutrient and oxygen delivery to fractures and discussed the potentially detrimental impact of adding excessive exogenous angiogenic and morphogenetic factors such as vascular endothelial growth factor, BMP-2, and BMP-4.^21^ The authors suggested that overapplication of morphogenetic factors may impede bone healing.

The etiology of our patient's nonunion was evaluated at each recurrence throughout his treatment course. Dorsalis pedis and tibialis posterior pulses were diminished but present and symmetric in the patient's bilateral lower extremities throughout the 7-year treatment course. Additionally, bone healing was evident on interval radiographs, and the patient reported improvement of his pain, motion, and mobility for periods of up to 16 months. During the third reconstruction, a nutrition panel demonstrated the patient did not have any nutritional deficiencies or clear indications causing nonunion. Surgeons involved in this case attempted to treat the fracture using a variety of mechanical and biologic interventions that continued to fail. An ABI test was not ordered until the patient's cardiology appointment in 2018. Obtaining an ABI earlier in treatment could have potentially helped detect and address the patient's vascular compromise sooner.

## CONCLUSION

This report documents a case of multiple reconstructions of a distal femur fracture that ultimately healed after a superficial femoral artery occlusion was addressed. In our literature search, we did not find any reports of distal femur fractures that healed following an endarterectomy of the superficial femoral artery. Recommendations for analyzing vascular health in patients with such injuries include performing an ABI as part of the physical examination for recalcitrant lower extremity nonunion and obtaining a transcutaneous oxygen measurement and a TBI. Developing research in the relationship between blood supply and fracture union, as well as in the use of morphogenetic factors, may help surgeons determine optimal treatment modalities for nonunion repair.

## Notes

*This article meets the Accreditation Council for Graduate Medical Education and the American Board of Medical Specialties Maintenance of Certification competencies for Patient Care and Medical Knowledge.*
